# Intraoperative Cerebral Hemodynamic Monitoring during Carotid Endarterectomy via Diffuse Correlation Spectroscopy and Near-Infrared Spectroscopy

**DOI:** 10.3390/brainsci12081025

**Published:** 2022-08-02

**Authors:** Kutlu Kaya, Alexander I. Zavriyev, Felipe Orihuela-Espina, Mirela V. Simon, Glenn M. LaMuraglia, Eric T. Pierce, Maria Angela Franceschini, John Sunwoo

**Affiliations:** 1 Optics at Athinoula A. Martinos Center for Biomedical Imaging, Department of Radiology, Massachusetts General Hospital, Harvard Medical School, Boston, MA 02114, USA; zav.shurik@gmail.com (A.I.Z.); f.orihuela-espina@bham.ac.uk (F.O.-E.); mfranceschini@mgh.harvard.edu (M.A.F.); 2 Department of Physiology, Faculty of Medicine, Hacettepe University, 06230 Ankara, Turkey; 3 School of Computer Science, University of Birmingham, Birmingham B15 2TT, UK; 4 Department of Neurology, Massachusetts General Hospital, Harvard Medical School, Boston, MA 02114, USA; mvsimon@mgh.harvard.edu; 5 Division of Vascular and Endovascular Surgery in the General Surgical Services, Massachusetts General Hospital, Harvard Medical School, Boston, MA 02114, USA; glamuraglia@mgh.harvard.edu; 6 Department of Anesthesia, Critical Care and Pain Medicine, Massachusetts General Hospital, Harvard Medical School, Boston, MA 02114, USA; etpierce@mgh.harvard.edu

**Keywords:** diffuse correlation spectroscopy, cerebral blood flow, near-infrared spectroscopy, carotid endarterectomy, intraoperative neuromonitoring, cerebral autoregulation

## Abstract

Objective: This pilot study aims to show the feasibility of noninvasive and real-time cerebral hemodynamic monitoring during carotid endarterectomy (CEA) via diffuse correlation spectroscopy (DCS) and near-infrared spectroscopy (NIRS). Methods: Cerebral blood flow index (CBF_i_) was measured unilaterally in seven patients and bilaterally in seventeen patients via DCS. In fourteen patients, hemoglobin oxygenation changes were measured bilaterally and simultaneously via NIRS. Cerebral autoregulation (CAR) and cerebrovascular resistance (CVR) were estimated using CBF_i_ and arterial blood pressure data. Further, compensatory responses to the ipsilateral hemisphere were investigated at different contralateral stenosis levels. Results: Clamping of carotid arteries caused a sharp increase of CVR (~70%) and a marked decrease of ipsilateral CBF_i_ (57%). From the initial drop, we observed partial recovery in CBF_i_, an increase of blood volume, and a reduction in CVR in the ipsilateral hemisphere. There were no significant changes in compensatory responses between different contralateral stenosis levels as CAR was intact in both hemispheres throughout the CEA phase. A comparison between hemispheric CBF_i_ showed lower ipsilateral levels during the CEA and post-CEA phases (*p* < 0.001, 0.03). Conclusion: DCS alone or combined with NIRS is a useful monitoring technique for real-time assessment of cerebral hemodynamic changes and allows individualized strategies to improve cerebral perfusion during CEA by identifying different hemodynamic metrics.

## 1. Introduction

Carotid endarterectomy (CEA) is a surgical procedure performed to reduce the risk of embolic stroke by removing the atherosclerotic plaque at the carotid bifurcation while providing normal cerebral blood flow (CBF). This procedure entails unilateral cross-clamping of the three branches of the carotid artery (common, internal, and external), leading to a complete cessation of blood flow [1]. During the cross-clamping, oxygen-rich blood is delivered to the ipsilateral hemisphere via collateral circulation, which originates from the posterior and the contralateral anterior circulation via the circle of Willis (CoW) [2,3]. While cerebral autoregulation (CAR) ensures stable CBF, there may not be adequate delivery and compensation due to abnormal CoW anatomy and/or autoregulatory disturbance of intracranial hemodynamics. Therefore, sudden CBF reductions or cerebral hypoperfusion can lead to neurophysiologic dysfunctions and potential neuronal cell death [4]. To avoid hypoperfusion, a shunt can be inserted through the clamped carotid arteries, which restore arterial inflow to the ipsilateral cerebral hemisphere. These are associated with a low incidence of carotid intimal injury and can encumber the exposure. However, this makes the procedure technically very challenging. Therefore, there is a need for a continuous, direct, and noninvasive monitoring of cerebral hemodynamics during CEA to facilitate timely interventions and prevention.

Currently, CEA patients are often monitored intraoperatively with somatosensory evoked potential (SSEP), electroencephalogram (EEG), carotid stump pressure, and Transcranial Doppler (TCD) ultrasound, which directly quantify the amount of cerebral hypoperfusion and its severity on neuronal function [4,5,6]. Since SSEP and EEG are noninvasive methods used to measure postsynaptic neuronal activity, they can show abnormal CBF changes as surrogate measures. However, various anesthetic agents and their doses affect neuronal activities that could suppress SSEP and EEG waveforms, making them difficult to rely on for ensuring adequate CBF [7]. Moreover, the utility of these neurophysiologic tests is dependent on the availability of expert clinical neurophysiologists and the use of certain anesthetic regimens that will allow reliable recordings. In addition, some patients’ postoperative course results in neurological deficits, even though no significant EEG changes were shown during the CEA procedure [6,8]. Unlike SSEP and EEG, carotid stump pressure and TCD ultrasound are perfusion-related measurements. Carotid stump pressure is measured at the distal end of the internal carotid artery after common and external carotid arteries are clamped. While indicating the collateral circulation efficiency of the CoW, carotid stump pressure is a noncontinuous measurement with limited utility [9]. TCD ultrasound can measure blood flow velocity reductions in the middle cerebral artery and detect emboli [10]. However, TCD ultrasound is not generally adopted for intraoperative CEA monitoring because of the difficulty maintaining alignment with the middle cerebral artery for long periods, the inability to measure the proximal part of the cerebral arteries, and the need for a skilled technician [11]. In addition, TCD ultrasound cannot be used on 10–15% of patients, as they lack a sufficient temporal bone window [12].

In recent years, optical neuroimaging techniques have made notable progress in providing easy-to-use and noninvasive hemodynamic monitoring devices to augment our understanding of cerebral hemodynamics during surgeries. Cerebral oximetry based on near-infrared spectroscopy (NIRS) uses light attenuation in the optical spectrum of 650–850 nm to quantify regional concentration changes in oxy-hemoglobin (HbO), deoxy-hemoglobin (HbR), total hemoglobin (HbT), and hemoglobin oxygenation (SO_2_) of the mixed arterial, capillary, and venous bed under the optical probe [13]. NIRS has been previously used in CEA to measure changes related to carotid clamping or stent insertion [14]. SO_2_ reductions correlate with changes in EEG, TCD ultrasound, carotid stump pressure, and postoperative neurologic deficits [5,15,16,17,18]. Furthermore, previous NIRS studies showed the ranges of sensitivity (30–80%) and specificity (77–98%) for brain ischemia detection, which is comparable to the sensitivity and specificity reached with the other modalities [19,20]. 

Diffuse correlation spectroscopy (DCS) is an emerging alternative optical technique able to directly measure an index of blood flow (BF_i_, cm^2^/s) by quantifying the temporal fluctuations in the intensity of the speckle pattern formed by coherent near-infrared light created by moving scatterers (e.g., erythrocytes) [21]. The time of these fluctuations is quantified by the intensity autocorrelation function (g_2_) from the detected light intensity. By fitting the correlation diffusion equation to the g_2_, DCS can quantify a BF_i_ where the faster decay of the correlation diffusion function indicates faster blood flow [22,23,24]. 

The perfusion index provided by DCS in conjunction with NIRS may provide critical information to the clinical team for preventing perioperative neurological injury and intraoperative complications during CEA. Therefore, this pilot study aimed to show the feasibility of using a combined DCS-NIRS system to noninvasively and continuously acquire cerebral BF_i_ (CBF_i_) and hemoglobin oxygenation. In addition, we estimated CAR indices in response to fluctuations in mean arterial blood pressure (MAP) and changes in cerebrovascular resistance (CVR) during CEA. Together, these measurements can protect the brain from hypo- or hyperperfusion and help to facilitate strategies for maximizing cerebral perfusion during CEA by accurately determining changes in cerebral hemodynamics. 

## 2. Materials and Methods

### 2.1. Study Patients and Procedure

**Figure 1:**
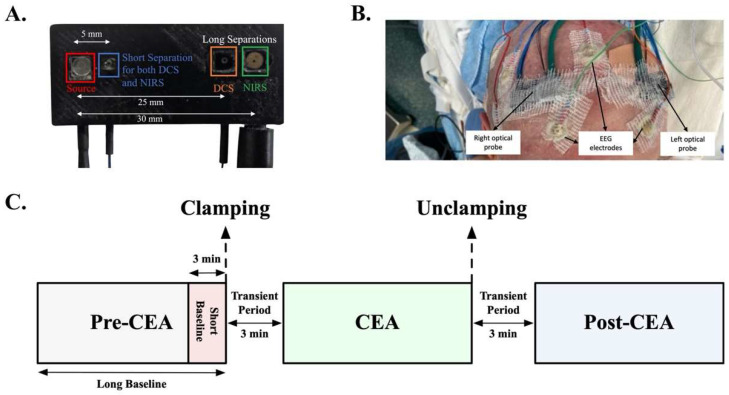
(**A**) The DCS-NIRS probe. The co-localized DCS and NIRS source fiber is marked by the red square. The co-localized DCS and NIRS short separation detector fiber (blue square) is located at 5 mm from the source. The short separation detector is employed to measure superficial (extracerebral) blood flow and hemoglobin oxygenation changes. A DCS long separation detector (orange square) is located at 25 mm (it includes three detectors to improve SNR), and a NIRS long separation detector (green square) is located at 30 mm from the source. Long separations are employed to measure cerebral blood flow and hemoglobin oxygenation. (**B**) The optical probes after bilateral placement on one patient in conjunction with EEG electrodes. (**C**) Schematic timeline of the surgery and data analysis phases. The signal average of a physiologically stable 3-min period immediately before clamping was used to normalize the data to quantify the transient ipsilateral changes of clamping and unclamping across patients (red shaded area, short baseline). The pre-CEA phase (gray and red shaded areas, long baseline) was used to normalize the data to quantify changes in the CEA (green shaded area) and post-CEA (blue shaded area) phases with respect to the pre-CEA phase. The 3 min of transient data between the two phases were discarded from the second analysis.

Twenty-six patients who underwent CEA between September 2016 and January 2021 were consented, twenty-four of whom (age 68 ± 8, range 53–90, 10 females) were included in this study. One patient was excluded due to a hardware issue, and one was excluded because the optical probe was placed on the temporal rather than a prefrontal area for evaluation of the signal quality at the very beginning of the study. Table 1 indicates the remaining patients’ demographics, measurement type, optical modality, measurement and CEA duration, degree of ipsi- and contralateral stenosis, shunt utilization, groups, and comorbid diseases. CEA patients complying with inclusion criteria were approached during their preoperative visit. The study was explained to the patients by a physician and the patients were given sufficient time to decide whether to participate and sign the informed consent. One or two optical probes (Figure 1A) were secured to the patient’s forehead alongside the clinically used EEG electrodes during preoperative procedures (Figure 1B). Measurements were taken in the operating room under general anesthesia from anesthesia induction until the end of anesthesia administration. Induction was carried out with sevoflurane in eighteen patients and with isoflurane in six patients. Anesthesia was also maintained mostly using propofol and in some patients with propofol and fentanyl.

In the first seven patients, we measured unilateral CBF_i_ via DCS on the forehead’s ipsilateral to the surgery side. As the study evolved, in the next three patients, we measured bilateral CBF_i_. In the remaining fourteen patients, we employed both DCS and NIRS bilaterally (Table 1, Measurement Type). For all DCS measurements, we used source-detector separations of 5 and 25 mm (except patient #10, who had separations of 5 and 30 mm) for the superficial and brain-sensitive measurements, respectively. For NIRS measurements, we used source-detector separations of 5 and 30 mm and analyzed the data using the modified Beer–Lambert law under pathlength and initial hemoglobin concentration assumptions [13]. During post-processing, patients were divided into three groups: (1) shunt utilization (SH group, *n* = 5), (2) moderate-to-substantial (≥50%) contralateral stenosis (SCS group, *n* = 5), and (3) normal-to-mild (<50%) contralateral stenosis (MCS group, *n* = 14) [25]. Since the ipsilateral hemisphere is mostly dependent on compensatory blood flow from the collateral circulation during clamping, the degree of contralateral stenosis was chosen for grouping. However, shunt utilization was separated into a different group, since there was supplemental blood supply to the ipsilateral hemisphere. The shunt utilization was decided by the clinical team intraoperatively in four patients (#5, #11, #20, and #22) due to immediate EEG changes and slowing down in fast frequencies after 1–2 min of clamping. On one patient (#10), the shunt decision was planned preoperatively, as the patient may have encountered hemodynamic challenges given the patient’s stroke history and cardiovascular diseases.

### 2.2. Optical Instrument and Clinical Auxiliary Signals

For the measurements, we used a frequency-domain NIRS-DCS system, MetaOx (ISS Inc., Champaign, IL, USA) [26]. The DCS component of the MetaOx system includes a long-coherence length laser at 850 nm and eight single-photon counting avalanche photodiode detectors, while the NIRS component includes eight diode lasers emitting at different wavelengths (λ) between 670 and 830 nm and four photomultiplier tube detectors. To achieve bilateral measurements, we added an external DCS system, which has a long-coherence length laser at 850 nm (CrystaLaser). We also divided the NIRS’ eight diode lasers into two probes: the right forehead probe with wavelengths of 672, 706, 759, and 830 nm, and the left forehead probe with wavelengths of 690, 726, 784, and 813 nm. To detect light at a short and long separation on each probe, one DCS detector detected light at 5 mm and three detected lights at 25 mm from the source. For NIRS in each probe, we used one detector at 5 and one at 30 mm from the source for short and long separations, respectively. The longer separation with NIRS, 30 mm instead of 25 mm, was chosen to account for the lower penetration sensitivity of NIRS with respect to DCS [27]. Figure 1A shows the optical probe geometry and Figure 1B shows the right and left probes arranged on a patient’s forehead. Clinical physiological recordings were co-acquired during our measurements, including electrocardiogram, EEG, and arterial blood pressure. EPIC electronic medical record system (Verona, Wis) was used to obtain other data, including pre-surgery Doppler ultrasound results and stenosis levels.

### 2.3. Analysis of Cerebral Blood Flow Index and Oxygenation

DCS intensity temporal autocorrelation function was acquired at 2 Hz for patients #1–10 and at 10 Hz for the remaining patients. In both cases, data were down-sampled to 0.2 Hz via a windowed averaging of g_2_ traces to enhance the signal-to-noise ratio (SNR). To obtain a BF_i_ signal, the g_2_ curves at each source-detector separation were fitted using the correlation diffusion equations for a semi-infinite medium [21] by assuming a fixed absorption coefficient (*µ*_a_ = 0.2 cm^−1^) and reduced scattering coefficient (*µ*_s_′ = 8.0 cm^−1^) for all subjects [28]. Co-recorded auxiliary signals were also resampled to 0.2 Hz. 

NIRS data from eleven patients (patients #11, 12, 13, 14, 17, 18, 19, 20, 21, 22, and 24) were used for this analysis. Three patients were excluded due to hardware issues (patients #15, 16, and 23). As in DCS, the raw NIRS intensity data were down-sampled to 0.2 Hz from 10 Hz via a windowed averaging. The changes in optical density (∆OD) were converted into changes in hemoglobin concentration (∆HbO, ∆HbR, and ∆HbT) using the modified Beer–Lambert law [13]. To fit the absorption coefficients at four wavelengths per probe, we first calculated absorption coefficients at each wavelength by using the extinction coefficients in the literature [29] with an assumed 75% concentration of water [30] and assumed initial HbO of 50 µMol, HbR of 30 µMol, and HbT of 80 µMol. The differential pathlength factor (DPF) was assumed at each wavelength following a scattering decay of *µ*_s_′ = 8.0 × (λ/850)^−1.5^ (see Supplementary Table S1 for *µ*_a_, *µ*_s_′, and DPF values). SO_2_ was also derived by combining NIRS measurements with assumed initial HbO and HbT, corresponding to an initial SO_2_ of 62.5%. 

Two distinct baselines were defined for analyzing the short-term and long-term changes in CBF_i_, HbT, and SO_2_ during CEA. A 3-min short baseline was used to quantify the transient ipsilateral changes due to clamping and unclamping. In this case, relative CBF_i_ (rCBF_i_, relative = response/baseline), ∆HbT, and changes in SO_2_ (∆SO_2_) were calculated with respect to the signals during a 3-min, physiologically stable period before clamping (Figure 1C, short baseline, red shaded area). On the other hand, the long baseline, which is the whole post-induction pre-CEA phase until clamping (Figure 1C, long baseline, grey and red shaded area), was used to quantify rCBF_i_, ∆HbT, and ∆SO_2_ in order to evaluate changes during the CEA and post-CEA surgery phases with respect to the pre-CEA phase.

### 2.4. Estimation of Cerebral Autoregulation and Cerebrovascular Resistance

We estimated the indices of dynamic CAR capacity by computing cross-correlation coefficients between CBF_i_ at 25 mm and MAP in each surgery phase. To isolate the signal fluctuation related to autoregulatory activities reported in Lee et al. [31], we chose a five-minute non-overlapping window for cross-correlation and took the average correlation found in each surgery phase. Nonparametric Spearman’s correlation was computed to avoid the bias from unintended signal artifacts/outliers in the signal [32]. A correlation coefficient (*r*) of 0 denotes intact autoregulation while a value of 1 denotes loss of autoregulation. To evaluate CAR, we set a threshold of *r* ≥ 0.3 for loss of autoregulation [33,34]. CVR was calculated as CVR = MAP/CBF_i_. The long baseline was used to normalize CVR data, followed by quantification of relative CVR (rCVR) changes during CEA and post-CEA phases with respect to the pre-CEA phase.

### 2.5. Statistical Analysis

The short- and long-term cerebral hemodynamic changes were investigated between groups during the different surgery interventions (Figure 1C). To analyze clamp- and unclamp-induced short-term transient changes in cerebral hemodynamics, all data were normalized by the short baseline. The Kruskal-Wallis test was used to compare transient changes (drop and overshoot) in rCBF_i_, ∆HbT, and ∆SO_2_ between groups. 

For the comparisons of long-term hemodynamic responses (rCBF_i_, rCVR, ∆HbT, and SO_2_), all data were normalized by the long baseline and compare changes in CEA and post-CEA phases with the pre-CEA phase. In the CAR analysis, all surgery phases were compared. For these comparisons, a two-way analysis of variance (ANOVA) test is performed to analyze between-group differences and hemispheric responses, followed by a Tukey post hoc test for pairwise comparisons. We also performed the nonparametric Friedman ranking test to assess within-group repeated measure differences in hemodynamic changes and CAR.

Quadratic regression analysis was performed to quantify the relationship between the MAP changes by a patient and the corresponding hemispheric CBF_i_ levels for each phase. Our quadratic regression model was *CBF_i_* = *ß*_0_ + *ß*_1_ × *MAP* + *ß*_2_ × *MAP*^2^. All data are presented as median with interquartile range (IQR) unless otherwise noted. For all tests, a *p*-value < 0.05 was considered statistically significant. 

## 3. Results

### 3.1. Intraoperative Hemodynamic Changes

**Figure 2:**
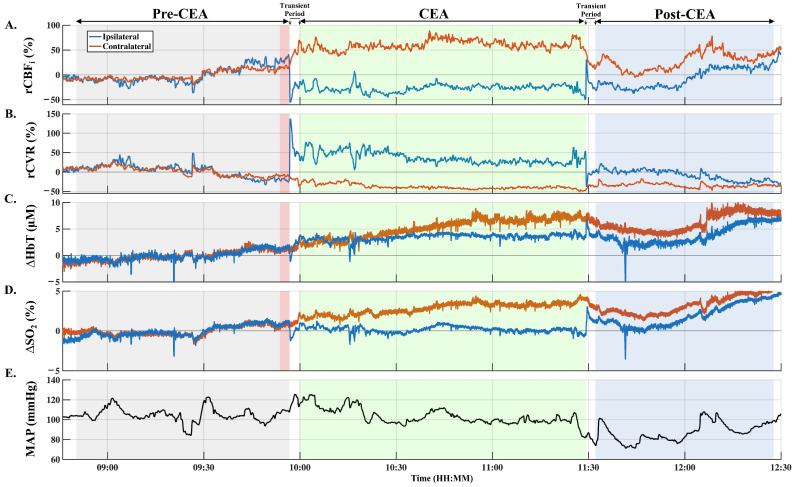
A typical measurement on a patient (#21, MCS group) during CEA. Ipsilateral and contralateral cerebral hemodynamic changes are shown in blue and orange, respectively, for rCBF_i_ (**A**), rCVR (**B**), ∆HbT (**C**), and ∆SO_2_ (**D**). MAP ((**E**), black) was measured invasively via an arterial cannula in the arm. All data are normalized with respect to the pre-CEA phase (long baseline), represented in grey and red shaded areas, except MAP. A three-minute short baseline is represented in the red shaded area. CEA and post-CEA phases are represented in shaded light green and blue areas, respectively. CEA, carotid endarterectomy; rCBF_i_, relative cerebral blood flow index; rCVR, relative cerebrovascular resistance; ∆SO_2_, changes in oxygen saturation; ∆HbT, changes in total hemoglobin concentration; MAP, mean arterial pressure.

The average duration of the surgery was 163 ± 43 min (mean ± SD, standard deviation), and the average CEA time was 58 ± 13 min. Figure 2 indicates a typical measurement from a patient (#21, MCS group). After clamping carotid arteries, ipsilateral rCVR increased sharply (Figure 2B, blue line), causing a steep and rapid decreased in ipsilateral rCBF_i_ (Figure 2A). The reductions in ipsilateral rCBF_i_ also caused a decrease of both ∆HbT and ∆SO_2_ (Figure 2C,D, respectively). Once ipsilateral rCBF_i_ reached a minimum value, it was weakly recovered over a short period and congregated to a new value lower than the pre-CEA baseline during the CEA phase (Figure 2A, green area). 

In the ipsilateral hemisphere, throughout the CEA phase, we observed an increase of blood volume, a small reduction in rCVR, and a slight increase of rCBF_i_ with respect to initial changes (Figure 2A–C, respectively). Additionally, an increase of ∆HbT on the contralateral side (Figure 2C, orange line) caused a decrease of contralateral rCVR and thus increased contralateral rCBF_i_ throughout the CEA phase (Figure 2A,B, respectively). Finally, ipsilateral ∆SO_2_ remained close to its baseline value, although the contralateral ∆SO_2_ was increased during the CEA phase due to the increase of the contralateral rCBF_i_ (Figure 2D). 

Upon unclamping, there was a marked overshoot in rCBF_i_ and ∆HbT on the ipsilateral side, leading to an increase of ∆SO_2_. After the overshoot, rCBF_i,_ and ∆HbT recovered to the initial baseline, but at different rates. rCBF_i_ recovered slowly (after 30 min), whereas ∆HbT reached its baseline more rapidly (Figure 2A,C, respectively, blue area). The slow recovery in rCBF_i_ was likely due to the medically reduced MAP by the anesthesia provider using vasodilators to reduce the risk of bleeding and reperfusion injury during the post-CEA phase.

### 3.2. Quantification of Transient Ipsilateral Changes in rCBF_i_, ∆HbT, and ∆SO_2_

**Figure 3:**
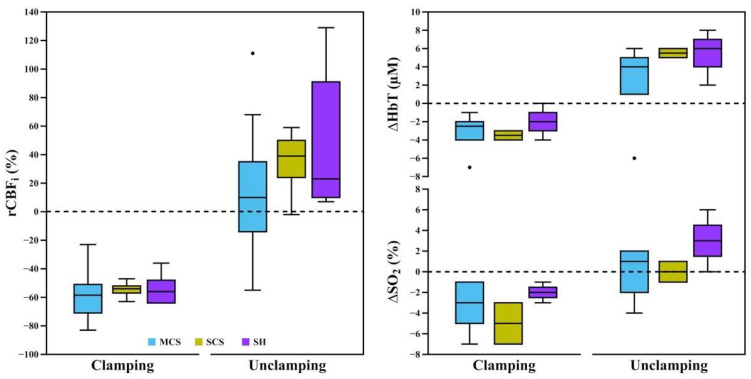
A quantified summary of short-term transient changes in clamping and unclamping in each group. rCBF_i_, ∆HbT, and ∆SO_2_ were quantified with respect to the short baseline. All data are represented in a whisker plot. Black-filled circles represent outliers. rCBF_i_, relative cerebral blood flow index; ∆HbT, changes in total hemoglobin concentration; ∆SO_2_, changes in oxygen saturation; MCS, normal-to-mild contralateral stenosis group (<50%); SCS, moderate-to-substantial contralateral stenosis group (≥50%); SH, shunt utilization group.

Figure 3 shows the average ipsilateral rCBF_i_, ∆HbT, and ∆SO_2_ transient changes at clamping and unclamping with respect to a short baseline for each group. Between-group differences were analyzed via the Kruskal-Wallis test and showed no statistical significance in rCBF_i_, ∆HbT, and ∆SO_2_ either at clamping or unclamping. However, the transient changes at unclamping were noticeably more elevated in the SCS and SH groups than in the MCS group. The average transient changes of ∆HbT and ∆SO_2_ at clamping were higher in the SCS group (median ∆HbT = −3.5 µM, ∆SO_2_ = −5%) than in other groups (MCS: ∆HbT = −2.5 µM, ∆SO_2_ = −3%; SH: ∆HbT = −2 µM, ∆SO_2_ = −2%). Across all patients, the averaged transient changes at clamping were −57 (IQR 15) % for rCBF_i_, −3 (2) µM for ∆HbT, and −3 (3) % for ∆SO_2_. The changes at unclamping were 21 (52) % for rCBF_i_, 5 (4) µM for ∆HbT, and 1 (3) % for ∆SO_2_.

### 3.3. Intraoperative Hemodynamic Changes during CEA and Post-CEA Phases

**Figure 4:**
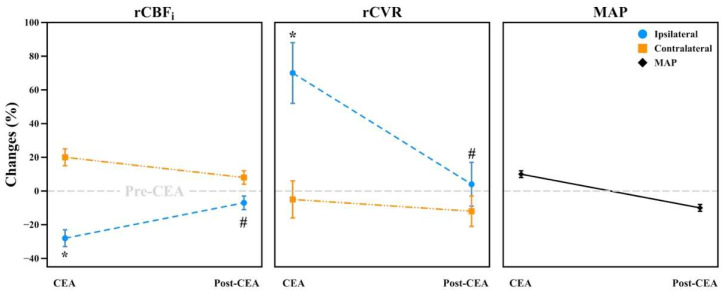
Hemodynamic changes in rCBF_i_, rCVR, and MAP of all patients during CEA and post-CEA phases. All changes were normalized with respect to the pre-CEA phase (long baseline, gray dashed line). All data are represented in mean ± standard error of the mean. Changes in the ipsilateral hemisphere were statistically significant than in the contralateral hemisphere both during CEA (indicated as *) and post-CEA phases (indicated as #). CEA, carotid endarterectomy; rCBF_i_, relative cerebral blood flow index; rCVR, relative cerebrovascular resistance; MAP, mean arterial pressure.

Between groups, rCBF_i_ and rCVR did not significantly differ in each phase (see Supplementary Figure S1). When averaging all patients, during the CEA and post-CEA phases, ipsilateral rCBF_i_ was significantly lower than contralateral rCBF_i_ (*F*_1,35_ = 39.42, *p* < 0.001 during CEA phase and *F*_1,35_ = 5.19, *p* = 0.03 during post-CEA phase) (Figure 4). Accordingly, ipsilateral rCVR was significantly higher than contralateral rCVR in the CEA (*F*_1,35_ = 24.89, *p* < 0.001) and post-CEA phases (*F*_1,35_ = 4.57, *p* = 0.04). MAP was elevated during the CEA phase (10 ± 12%) and was reduced (11 ± 10%) during the post-CEA phase with respect to the pre-CEA phase. ∆HbT and ∆SO_2_ did not significantly differ during CEA and post-CEA phases for all subjects (not shown).

### 3.4. Relationship between CBF_i_ and MAP, and Dynamic CAR Changes

**Figure 5:**
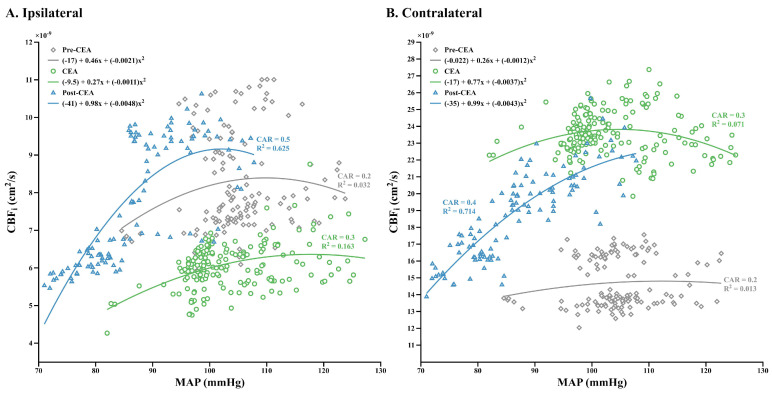
A representative case of ipsilateral (**A**) and contralateral (**B**) CBF_i_ versus MAP in a patient (#21, MCS group). In the ipsilateral hemisphere, there was a decrease of CBF_i_ for MAP below 85 mmHg during the post-CEA phase, suggesting the loss of cerebral autoregulation for these MAP values. In contrast, during the pre-CEA and CEA phases, CBF_i_ did not increase despite MAP being above 120 mmHg, as this patient was still in the autoregulatory range. The pre-CEA, CEA, and post-CEA phases are represented as grey diamond, green dotted, and blue triangle symbols, respectively. CAR values are the computed cross-correlation coefficients with respect to our method. The least-squares method is used to find a polynomial fit of the maximum degree of two and R^2^ values are the results of curve fitting. The fit results are shown in color with respect to phases. CBF_i_, cerebral blood flow index; MAP, mean arterial pressure; CEA, carotid endarterectomy; CAR, cerebral autoregulation.

A scatter plot from one patient (#21, MCS group) illustrates the relationship between CBF_i_ and MAP during the pre-CEA, CEA, and post-CEA phases in the ipsilateral and contralateral hemispheres (Figure 5A and B, respectively). Overall, ipsilateral CBF_i_ and MAP were more correlated in this patient during the post-CEA phase (CAR = 0.5, R^2^ = 0.625) than the CEA (CAR = 0.3, R^2^ = 0.163) and pre-CEA phases (CAR = 0.2, R^2^ = 0.032, Figure 5A). Our regression model showed that MAP accounted for 62.5% variability in ipsilateral CBF_i_ during the post-CEA phase, which aligns with the loss of CAR indicated by a value of *r* = 0.5. In a similar line, contralateral CBF_i_ and MAP were more correlated during the post-CEA phase (CAR = 0.4, R^2^ = 0.714) than the CEA (CAR = 0.3, R^2^ = 0.071) and pre-CEA phases (CAR = 0.2, R^2^ = 0.013, Figure 5B). On the contralateral side, MAP accounted for 71.4% variability in CBF_i_ during the post-CEA phase, and loss of CAR was indicated by a value of *r* = 0.4.

**Figure 6:**
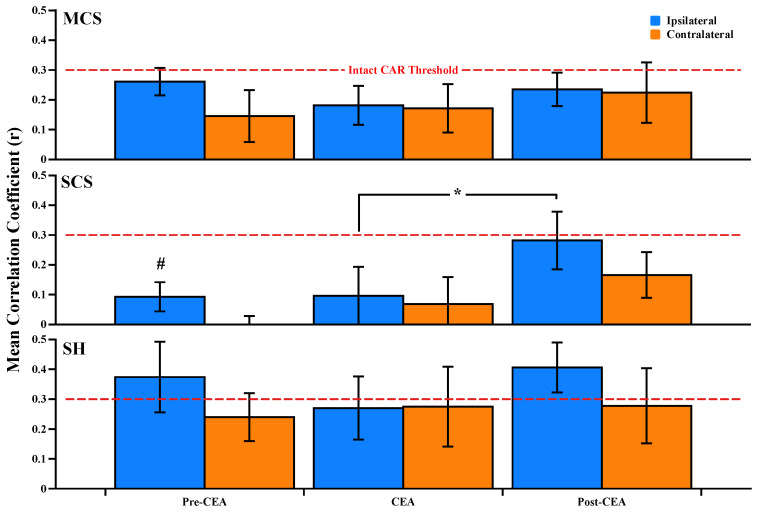
Mean correlation coefficients (*r*) between CBF_i_ and MAP during pre-CEA, CEA, and post-CEA phases in the MCS, SCS, and SH groups. The ipsilateral hemisphere is blue, and the contralateral hemisphere is represented in orange color. CBF_i_ was more autoregulated in the SCS group than MCS and SH groups during the pre-CEA phase (indicated as #, *p* = 0.003). The intact CAR threshold (*r* = 0.3) is indicated as a red dashed line. Data are shown as mean ± standard error of the mean. * *p* = 0.03. CAR, cerebral autoregulation; CBF_i_, cerebral blood flow index; MAP, mean arterial pressure; CEA, carotid endarterectomy; MCS, mild contralateral stenosis group (<50%); SCS, substantial contralateral stenosis group (≥50%); SH, shunt group.

During the pre-CEA phase, there were significant differences between groups (*F*_2,35_ = 6.34, *p* < 0.05) and between the hemispheres (*F*_1,35_ = 4.01, *p* < 0.05) in the CAR correlation coefficients. A Tukey post hoc test revealed that CBF_i_ was more autoregulated in the SCS group (CAR = 0.1 ± 0.05) than in the MCS (CAR = 0.26 ± 0.05) and SH groups (CAR = 0.37 ± 0.12, *p* = 0.003) during the pre-CEA phase (Figure 6). When combining all groups to investigate hemispheric differences, we found that the contralateral hemisphere (CAR = 0.12 ± 0.05) was also more autoregulated than the ipsilateral hemisphere (CAR = 0.24 ± 0.04, *p* = 0.04) during the pre-CEA phase (see Supplementary Figure S2). In the SCS group, the Friedman test revealed that ipsilateral CBF_i_ showed significant autoregulatory responses during the CEA phase (CAR = 0.1 ± 0.1) than in the post-CEA phase (CAR = 0.28 ± 0.1, *p* = 0.03, Figure 6). Lastly, the loss of ipsilateral CAR indicated by a value of *r* ≥ 0.3 was seen in the SH group both during the pre-CEA (CAR = 0.37 ± 0.12) and post-CEA phases (CAR = 0.41 ± 0.08).

### 3.5. Perioperative Complications

Our patients tolerated the procedure well and their full operative course remained neurologically intact, with no signs of transient ischemic attack (TIA) or stroke. However, the postoperative course of some patients was notable for mild headache, hypotension, or hypertension. During the postoperative course, these symptoms were resolved without issues. In two patients (#4 and #12), there was a weakness in the left lower extremity. There were also postoperative transient neurological changes in two patients (#4 and #24) who had a slightly asymmetric smile. These improved throughout the patients’ stay and the patients were discharged in stable condition with no new neurologic deficits. Furthermore, 30-day follow-ups of our patients reported no sign of symptoms of TIA or stroke. Additionally, their blood pressure was well controlled.

In summary, we found that both ipsilateral CEA and post-CEA CBF_i_ levels were significantly lower than that of contralateral due to significantly higher CVR levels. Furthermore, CAR analysis showed that CBF_i_ was more significantly autoregulated in the SCS group than in other groups during the pre-CEA phase. In the SCS group, although CAR was intact during the post-CEA phase, ipsilateral CBF_i_ was significantly less autoregulated than in the CEA phase. 

## 4. Discussion

This is the first study that utilized CAR and CVR estimates from DCS-derived CBF_i_ and MAP measurements during the CEA procedure. This pilot study also demonstrated (1) the feasibility of noninvasive CBF monitoring during CEA, acquired by DCS alone and in combination with NIRS, (2) the transient cerebral hemodynamic changes due to clamping and unclamping of carotid arteries, and (3) hemodynamic compensation in different degrees of contralateral stenosis and shunt utilization.

### 4.1. Clamp- and Unclamp-Induced Transient Hemodynamic Changes

Both clamping and unclamping caused marked changes in cerebral hemodynamics. In the ipsilateral hemisphere, clamping caused a mechanical increase of CVR, and thus, a transient interruption of perfusion, resulting in a rapid drop of CBF_i_ (Figure 2A,B, respectively). This decreased perfusion triggered an increase of ∆HbT (i.e., blood volume) and provided a partial recovery of CBF_i_ (Figure 2A,C, respectively). Conversely, unclamping caused a marked overshoot of CBF_i_, ∆HbT, and ∆SO_2_ (Figure 2A,C,D, respectively) due to a rapid drop of CVR (Figure 2B). 

There were also marked changes in the contralateral hemisphere due to clamping and unclamping. Medically elevated MAP at clamping contributed to an increase of blood volume and CBF_i_ (Figure 2A,E, respectively). This contralaterally increased CBF_i_ at clamping also contributed to the aforementioned recovery to ipsilateral CBF_i_ due to vascular pressure difference. Upon unclamping, while MAP was medically reduced, there was a decrease of contralateral CBF_i_, ∆HbT, and ∆SO_2_ due to the gradual recovery of vascular pressure (Figure 2A,C–E, respectively). However, these transient changes due to clamping and unclamping did not reveal a significant difference between groups, indicating adequate collateral compensation for our patients (Figure 3). It is also worth noting that clamping of ipsilateral carotid arteries produced changes in the EEG activity with attenuation of amplitude and fast frequencies for four patients in the SH group, which led to the decision to place a shunt intraoperatively. Therefore, the lack of difference in the SH group might be due to shunt-provided supplemental blood flow. 

### 4.2. Intraoperative Changes in CBF_i_ and CVR

During the CEA phase, ipsilateral CVR was significantly higher than that of the contralateral due to additional clamp-induced resistance (Figure 4, *p* < 0.001). Therefore, the average ipsilateral CBF_i_ was significantly lower than contralateral (Figure 4, *p* < 0.001). A previous study reported that both ipsilateral and contralateral CBF_i_ was lower than their baseline values during the CEA phase [35]. We found a different response in the contralateral CBF_i_, which was higher than its baseline value. This might be due to several reasons such as contralateral flow compensation, the presence of communicating arteries, and thus, the integrity of CoW, and MAP management during the procedure. Nevertheless, an increase of contralateral CBF_i_ would be expected during unilateral clamping as it provides quantitatively significant compensative blood flow through CoW [36]. Our results showed an adequate collateral compensation by means of increased contralateral CBF_i_ in our patients. However, the ipsilateral CBF_i_ levels were different between groups. There was a lower ipsilateral CBF_i_ in the SCS and MCS groups, indicating the limited collateral compensation due to contralateral stenosis. In the SH group, however, the shunt provided supplemental blood flow, and therefore, the ipsilateral CBF_i_ reduction was lower than in other groups (see Supplementary Figure S1).

During the post-CEA phase, we observed that averaged ipsilateral CBF_i_ recovered gradually to its baseline. However, this ipsilateral CBF_i_ was significantly lower than that of the contralateral (Figure 4, *p* = 0.03). This was mainly due to the presence of high ipsilateral CVR. Interestingly, we observed that ipsilateral CBF_i_ was higher than the baseline in the SH group, demonstrating the efficacy of shunt utilization, as the additional blood flow can help in recovery (see Supplementary Figure S1).

### 4.3. Dynamic CAR Indices

During the pre-CEA phase, we observed that the highest correlation greater than 0.3 was in the SH group on the ipsilateral hemisphere (Figure 6, SH group). Since the shunt utilization was decided either intraoperatively based on EEG changes or pre-surgically based on the patient’s stroke history and neurological evaluation, ipsilaterally weakened CAR in the SH group during this phase may reflect the degrees of their past ischemic stroke or transient ischemic attacks [37,38]. The better CAR in the SCS group than in other groups during the pre-CEA phase may actually be an indication of the ability to maintain constant CBF_i_ for elevated MAP (Figure 6, *p* = 0.003). Regardless, we observed significantly worsened CAR in the ipsilateral hemisphere compared to the contralateral hemisphere during the pre-CEA phase (see Supplementary Figure S2).

During the CEA phase, CAR was intact in both hemispheres for each group, as CAR curves were still in the autoregulatory range, as we demonstrated in Figure 5A,B, respectively. During the post-CEA phase, worsened CAR was found in the SH group, which may be due to an acute inability to adapt to removing supplemental blood flow and thus tightly controlled MAP. Furthermore, in general, CAR was not improved immediately in the post-CEA phase, which was probably due to the intraoperatively reduced MAP to avoid hyperperfusion syndrome (see post-CEA phase in Figure 2E and the high correlation between CBF_i_ and MAP at 70–90 mmHg in Figure 5A,B). Nevertheless, the CAR estimates in the post-CEA phases might be impacted by medications to control patients’ arterial blood pressure.

### 4.4. Hemodynamic Changes in the Superficial Compartment

Most patients showed similar hemodynamic trends in the extracerebral measurements (detected at 5 mm) throughout the CEA procedure: significant decreases in the ipsilateral superficial BF_i_ (SBF_i_) and oxygenation upon clamping, followed by a small recovery during CEA (see Supplementary Figure S3). These changes in SBF_i_ are consistent with the procedural clamping of the external carotid artery, and the partial recovery of SBF_i_ is probably due to perfusion via collateral circulation through the ophthalmic artery [39].

### 4.5. Limitations

As limitations, we are intrinsically limited by NIRS and DCS, as our model assumes consistent head anatomy and no extracerebral interference differences across subjects. One limitation of our study is the small number of patients enrolled for CBF_i_ and NIRS comparisons between groups. Another limitation of our study is the assumption of the same optical properties among all patients. These limitations may contribute to the inter-subject variability in CBF_i_ and ∆HbT. However, many other factors could lead to such variations in cerebral hemodynamics, including the severity of the bilateral stenosis, comorbid diseases, smoking status, blood pressure, heart rate, anesthetics, and cerebral adaptive capacity. In particular, different anesthetic regimens produce different reductions in cerebral blood flow and metabolic rate due to tight coupling. This is in contrast to the flow-metabolism decoupling in some anesthetic regimens, for example, propofol with higher concentrations of sevoflurane [40]. Since our study is not a clinically controlled study, we did not aim to investigate the dosage and combination of anesthetics. Nevertheless, most of our patients showed similar hemodynamic trends throughout the CEA procedure.

## 5. Conclusions

In conclusion, a combined optical DCS-NIRS system can provide important insight into cerebral hemodynamics during CEA, preventing undesirable events in ischemia-sensitive organs such as the brain. Since DCS is sensitive to changes in blood flow, intraoperative monitoring of clamping-induced cerebral hypoperfusion and unclamping-induced reperfusion would help make acute management changes in inpatient care (e.g., arterial shunting) to avoid brain tissue damage. Moreover, by monitoring CBF_i_ and DCS-derived indices, such as CAR (which was mostly intact throughout the surgery phases), our dataset can provide additional information about brain health. Thus, we conclude that DCS alone or combined with NIRS can provide complementary cerebral hemodynamic assessment during surgery, which can potentially guide the clinical team to minimize perioperative stroke and neurological injury by determining personalized perfusion strategies.

## Figures and Tables

**Table 1:** Measurement and patients’ clinical characteristics.

Patient	Sex	Measurement Type	Optical Modality	Measurement Duration	Clamp Duration	Ipsilateral Stenosis	Contralateral Stenosis	Shunt	Group
1	F	Unilateral	DCS	2 h, 52 min	48 min	70–89%	No stenosis	No	MCS
2	M	Unilateral	DCS	2 h, 29 min	53 min	70–89%	1–19%	No	MCS
3	F	Unilateral	DCS	2 h, 20 min	1 h, 4 min	20–49%	No stenosis	No	MCS
4	F	Unilateral	DCS	3 h, 58 min	1 h, 19 min	70–89%	20–49%	No	MCS
5	M	Unilateral	DCS	2 h, 19 min	1 h, 1 min	90–99%	1–19%	Yes	SH
6	M	Unilateral	DCS	3 h, 53 min	40 min	1–19%	1–19%	No	MCS
7	M	Unilateral	DCS	3 h, 17 min	1 h, 17 min	20–49%	1–19%	No	MCS
8	M	Bilateral	DCS	3 h, 34 min	58 min	70–89%	70–99%	No	SCS
9	F	Bilateral	DCS	3 h, 14 min	52 min	70–89%	No stenosis	No	MCS
10	F	Bilateral	DCS	2 h, 48 min	55 min	70–89%	1–19%	Yes	SH
11	M	Bilateral	DCS-NIRS	2 h, 48 min	1 h, 5 min	70–89%	20–49%	Yes	SH
12	M	Bilateral	DCS-NIRS	2 h, 25 min	1 h, 3 min	70–89%	No stenosis	No	MCS
13	F	Bilateral	DCS-NIRS	1 h, 35 min	37 min	70–89%	No stenosis	No	MCS
14	F	Bilateral	DCS-NIRS	2 h, 27 min	1 h, 3 min	20–49%	1–19%	No	MCS
15	F	Bilateral	DCS-NIRS	2 h, 33 min	49 min	70–89%	1–19%	No	MCS
16	M	Bilateral	DCS-NIRS	1 h, 53 min	46 min	50–69%	100%	No	SCS
17	M	Bilateral	DCS-NIRS	2 h, 47 min	1 h, 1 min	70–89%	1–19%	No	MCS
18	M	Bilateral	DCS-NIRS	2 h, 55 min	1 h, 4 min	70–89%	20–49%	No	MCS
19	F	Bilateral	DCS-NIRS	2 h, 11 min	43 min	70–89%	50–69%	No	SCS
20	F	Bilateral	DCS-NIRS	2 h, 24 min	56 min	50–69%	1–19%	Yes	SH
21	M	Bilateral	DCS-NIRS	4 h, 4 min	1 h, 34 min	70–89%	No stenosis	No	MCS
22	M	Bilateral	DCS-NIRS	2 h, 57 min	1 h, 4 min	50–69%	50–69%	Yes	SH
23	M	Bilateral	DCS-NIRS	1 h, 15 min	53 min	70–89%	70–89%	No	SCS
24	M	Bilateral	DCS-NIRS	2 h, 2 min	56 min	70–89%	50–69%	No	SCS
				MCS (*n* = 14)		SCS (*n* = 5)		SH (*n* = 5)	
Age, years (mean ± SD) Range				69 ± 10 53–90		69 ± 5 64–74		65 ± 2 62–68	
**Comorbid Disease**									
Hypertension				71%		80%		100%	
Diabetes-Mellitus				50%		20%		20%	
Coronary Disease				35%		0%		20%	
Hyperlipidemia				50%		40%		40%	
Afib				21%		0%		0%	

F, female; M, male; DCS, diffuse correlation spectroscopy; NIRS, near-infrared spectroscopy; CEA, carotid endarterectomy; SH, shunt; SCS, moderate-to-substantial (≥50%) contralateral stenosis; MCS, normal-to-mild (<50%) contralateral stenosis; Afib, atrial fibrillation; SD, standard deviation.

## Data Availability

The data sets used and/or analyzed for this study are available from the corresponding authors upon reasonable request.

## Supplementary Materials

The following supporting information can be downloaded at: https://www.mdpi.com/article/10.3390/brainsci12081025/s1, Table S1: Calculated µa, µs’, and DPF values in each wavelength for the right and left forehead probe. Figure S1: Hemodynamic changes in rCBF_i_, rCVR_,_ and MAP in each group during the CEA and post-CEA phases. Figure S2: Mean correlation coefficients (r) between hemispheres during the pre-CEA phase. Figure S3: A typical measurement on a patient (#21, MCS group) at 5 mm during CEA.
