# Supplementary material for: Intraoperative Cerebral Hemodynamic Monitoring during Carotid Endarterectomy via Diffuse Correlation Spectroscopy and Near-Infrared Spectroscopy
Source: Brain Sci. 2022 Aug 2;12(8):1025. doi: 10.3390/brainsci12081025 (PMC9405597; doi:10.3390/brainsci12081025)
Supplement: Supplementary file 1 [file brainsci-12-01025-s001.zip › brainsci-1828792-supplementary.pdf]

Supplemental Information for

Intraoperative Cerebral Hemodynamic Monitoring during Carotid Endarterectomy via Diffuse Correlation Spectroscopy and Near-Infrared Spectroscopy

Kutlu Kaya [1,2,*], Alexander I. Zavriyev [1], Felipe Orihuela-Espina [1,3], Mirela V. Simon [4], Glenn M. LaMuraglia [5], Eric T. Pierce [6], Maria Angela Franceschini [1] and John Sunwoo [1,*]

[1] Optics at Athinoula A. Martinos Center for Biomedical Imaging, Department of Radiology, Massachusetts General Hospital, Harvard Medical School, Boston, MA 02114, USA; zav.shurik@gmail.com (A.I.Z.); mfranceschini@mgh.harvard.edu (M.A.F.)
[2] Department of Physiology, Faculty of Medicine, Hacettepe University, 06230 Ankara, Turkey
[3] School of Computer Science, University of Birmingham, Birmingham B15 2TT, UK; f.orihuela-espina@bham.ac.uk
[4] Department of Neurology, Massachusetts General Hospital, Harvard Medical School, Boston, MA 02114, USA; mvsimon@mgh.harvard.edu
[5] Division of Vascular and Endovascular Surgery in the General Surgical Services, Massachusetts General Hospital, Harvard Medical School, Boston, MA 02114, USA; glamuraglia@mgh.harvard.edu
[6] Department of Anesthesia, Critical Care and Pain Medicine, Massachusetts General Hospital,
Harvard Medical School, Boston, MA 02114, USA; etpierce@mgh.harvard.edu
* Correspondence: kkaya@mgh.harvard.edu (K.K.); jsunwoo@mgh.harvard.edu (J.S.)

Supplementary Table S1. Calculated μa, μs′, and DPF values in each wavelength for the right and left forehead probe.

Forehead Probe	λ (nm)	μa (cm^{-1})	μs′ (cm^{-1})	DPF
**Right**	672	0.26	11.38	5.78
	706	0.17	10.57	6.77
	759	0.21	9.48	5.84
	830	0.19	8.29	5.60
**Left**	690	0.20	10.94	6.41
	726	0.16	10.13	6.87
	784	0.18	9.03	6.19
	813	0.18	8.55	6.01

λ, wavelength; *μa*, absorption coefficient; *μ*s′, reduced scattering coefficient; *DPF*, differential pathlength factor.

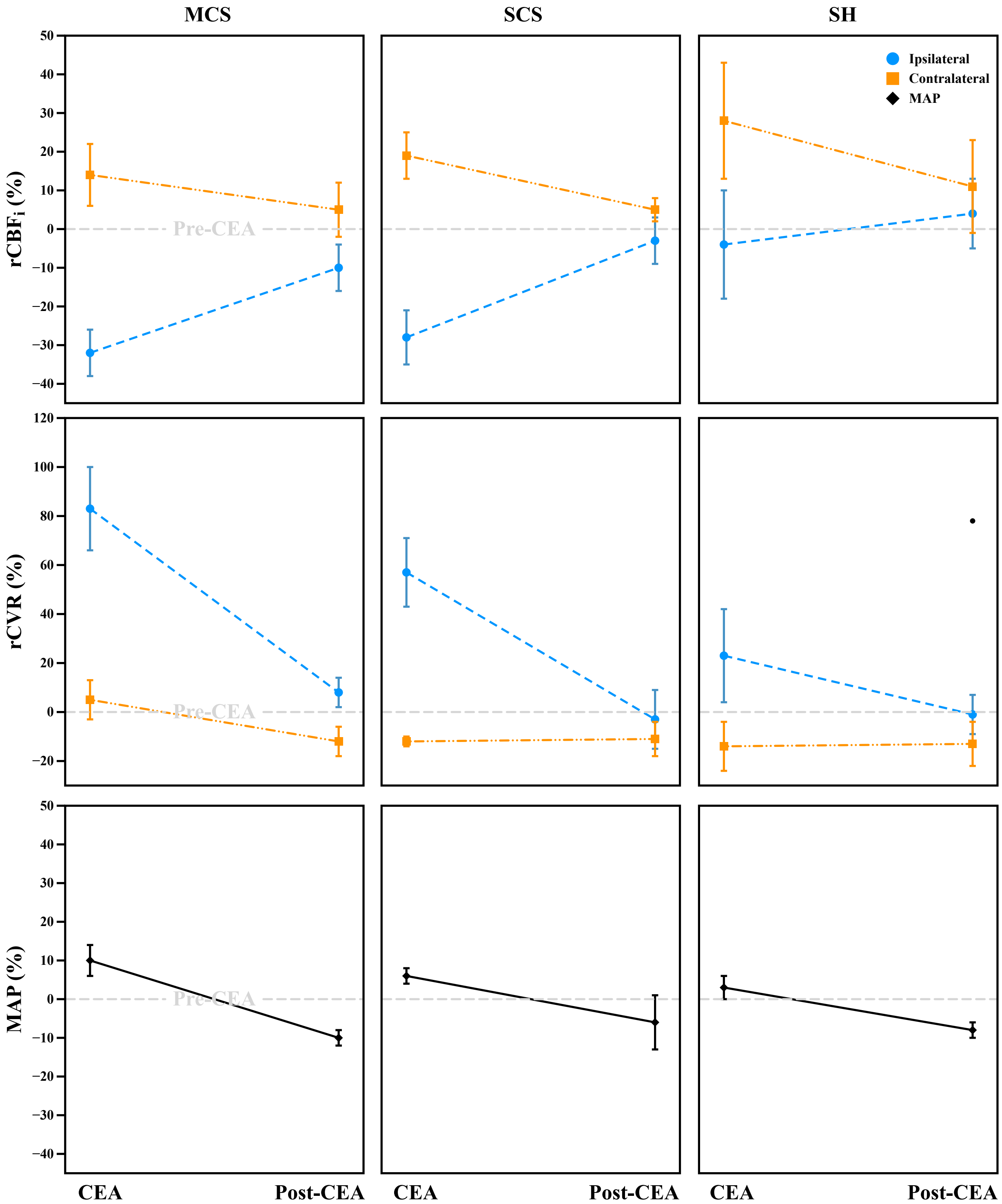

Supplementary Figure S1. Hemodynamic changes in rCBFi, rCVR, and MAP in each group during the CEA and post-CEA phases. All changes were normalized with respect to the pre-CEA phase (long baseline, grey dashed line). All data are represented in mean ± standard error of the mean. One patient was defined as an outlier in the SH

group, indicated as a black-filled circle. MCS, normal-to-mild contralateral stenosis group; SCS, moderate-to-substantial contralateral stenosis group; SH, shunt utilization group; CEA, carotid endarterectomy; rCBF$_i$, relative cerebral blood flow index; rCVR, relative cerebrovascular resistance; MAP, mean arterial pressure.

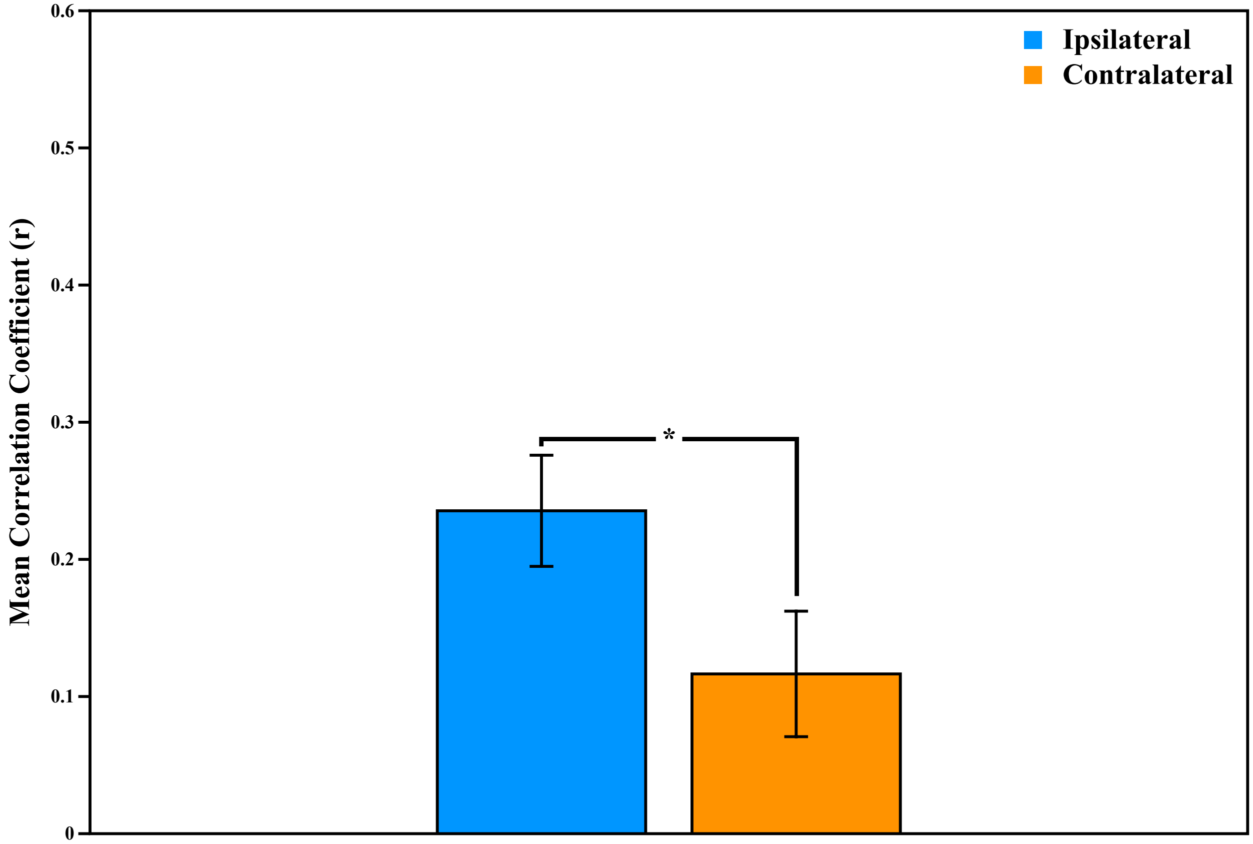

Supplementary Figure S2. Mean correlation coefficients (r) between hemispheres during the pre-CEA phase. When all patients are considered, CBF$_i$ was more autoregulated in the contralateral hemisphere than the ipsilateral hemisphere during the pre-CEA phase. * $p < 0.05$. CBF$_i$, cerebral blood flow index; MCS, normal-to-mild contralateral stenosis group; SCS, moderate-to-substantial contralateral stenosis group; SH, shunt utilization group.

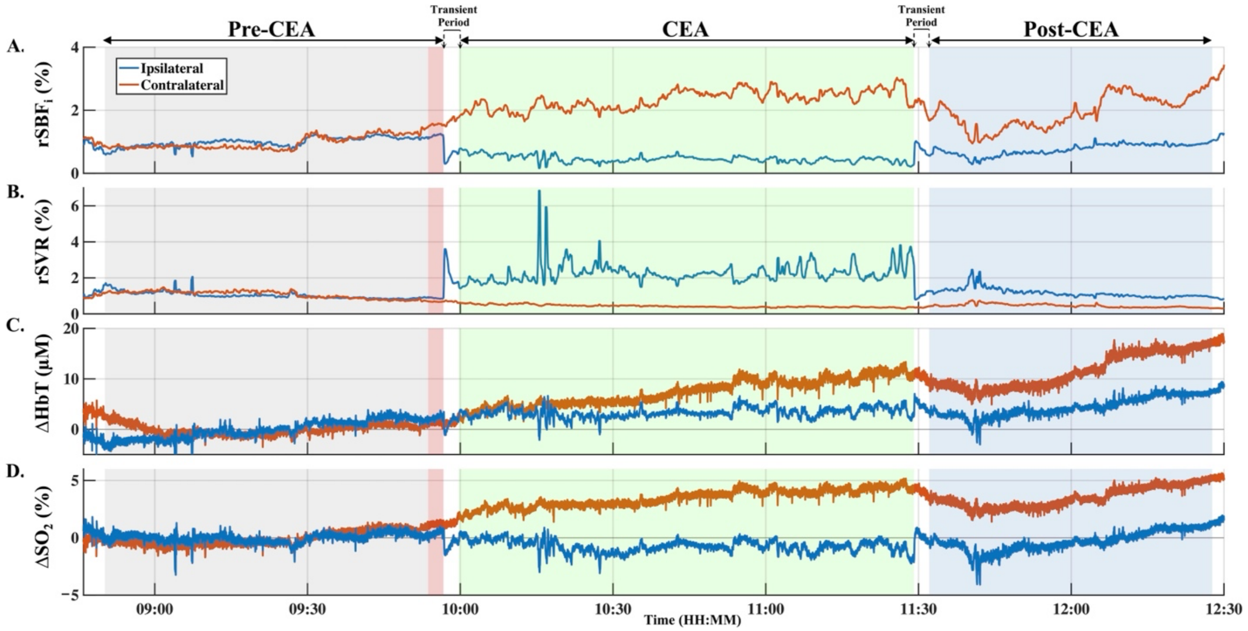

Supplementary Figure S3. A typical measurement on a patient (#21, MCS group) at 5 mm during CEA. All data are normalized with respect to the pre-CEA phase (long baseline), represented in grey and red shaded areas. Ipsilateral and contralateral hemodynamic changes at 5 mm are shown in blue and orange, respectively, for $rSBF_i$ (A), rSVR (B), ΔHbT (C), and ΔSO_2 (D). All data are normalized with respect to the pre-CEA phase (long baseline), represented in grey and red shaded areas. A three-minute short baseline is represented in the red shaded area. CEA and post-CEA phases are represented in shaded light green and blue areas, respectively. CEA, carotid endarterectomy; $rSBF_i$, relative superficial blood flow index; rSVR, relative superficial vascular resistance; ΔHbT, changes in total hemoglobin concentration; ΔSO_2, changes in oxygen saturation.
